# Screening for Familial Hypercholesterolemia in Children: What Can We Learn From Adult Screening Programs?

**DOI:** 10.3390/healthcare3041018

**Published:** 2015-10-26

**Authors:** Lidewij Henneman, Colleen M. McBride, Martina C. Cornel, Debra Duquette, Nadeem Qureshi

**Affiliations:** 1 Department of Clinical Genetics, Section of Community Genetics, EMGO Institute for Health and Care Research, VU University Medical Center, P.O. Box 7057, Amsterdam 1007 MB, The Netherlands; E-Mail: mc.cornel@vumc.nl; 2 Department of Behavioral Sciences and Health Education, Rollins School of Public Health, Emory University, Atlanta, GA 30322, USA; E-Mail: cmmcbri@emory.edu; 3 Genomics and Genetic Disorders Section, Michigan Department of Health and Human Services, Lansing, MI 48909, USA; E-Mail: duquetted@michigan.gov; 4 Division of Primary Care, School of Medicine, University of Nottingham, University Park, Nottingham NG7 2RD, UK; E-Mail: Nadeem.Qureshi@nottingham.ac.uk

**Keywords:** population screening, familial hypercholesterolemia, prevention, genetic testing, pediatrics, public health genomics

## Abstract

Familial hypercholesterolemia (FH), an autosomal dominant atherosclerotic disease, is a common monogenic subtype of cardiovascular disease. Patients with FH suffer an increased risk of early onset heart disease. Early identification of abnormally elevated cholesterol signpost clinicians to interventions that will significantly decrease risk of related morbidity and mortality. Cascade genetic testing can subsequently identify at-risk relatives. Accordingly, a number of screening approaches have been implemented for FH in countries including the UK and the Netherlands. However, incomplete identification of cases remains a challenge. Moreover, the potential for early intervention is now raising questions about the value of implementing universal cholesterol screening approaches that focus on children. In this report, we briefly discuss the potential benefit of such screening. Additionally, we submit that ever increasing genome technological capability will force a discussion of including genetic tests in these screening programs. We discuss the opportunities and challenges presented by such an approach. We close with recommendations that the success of such screening endeavors will rely on a better integrated practice model in public health genomics that bridges stakeholders including practitioners in primary care, clinical genetics and public health.

## 1. Introduction

Burgeoning rates of chronic disease and concordant escalation in health costs are of international concern. Accordingly, health organizations around the world are considering how best to direct limited public resources to address these challenges. In his seminal article “Sick individuals and sick populations,” Rose was among the first to argue that there might be greater public health benefit to risk reduction (and implicitly health cost containment) by targeting prevention efforts to a proportionately small, high risk subgroup than pursuing modest risk reduction across an entire population [1]. Most recently, ongoing advances in genome sequencing promise to enable identification of subgroups with monogenic hereditary syndromes [2] who may be an important target audience for this endeavor, especially for identifying healthy relatives at risk of preventable morbidity [3]. These family members could benefit from “precision medicine” approaches wherein treatments are customized and differ from that provided to groups with multifactorial common diseases [4].

One of the most common of these monogenic disorders is familial hypercholesterolemia (FH), an autosomal dominant atherosclerotic disease. Up to 1 in 200–500 individuals carry one abnormal gene associated with FH [5,6]. Worldwide, most individuals affected by FH have a single mutation (heterozygotic) in either the low density lipoprotein receptor (LDLR), apolipoprotein B (APOB), or proprotein convertase subtilisin/kexin type 9 (PCSK9) genes [5]. Homozygous FH is more rare and serious, with an estimated frequency of one in a million [6]. In both cases, individuals with FH suffer an increased risk of early-onset coronary heart disease (CHD). CHD will develop in approximately 50% of men by the age of 50, and 30% of women by the age of 60, resulting in a nearly 100-fold increase in mortality risk compared to the general population [7]. However, early identification of the condition, followed by lipid lowering treatment, can result in a 48% reduction of CHD mortality [8]. Accordingly, a prospective study showed that, after 10 years of statin treatment, the risk of myocardial infarction among those with FH did not differ from the general population [9].

Those at increased risk for FH can be identified by raised cholesterol (*i.e.*, high levels of cholesterol >7.5 mmol/L) and/or personal or family history of early onset CHD [10,11,12]. Despite the availability of these risk assessments, many individuals with FH are not identified and remain undiagnosed. For example, in the United Kingdom (UK), it has been estimated that up to 85% of individuals with FH are not identified [12].

Organized screening approaches for FH can be “targeted” (selective) to individuals that meet pre-specified risk criteria (e.g., those who have a positive family history for early CHD) or “universal” screening that involves evaluating all individuals in a particular segment of a population (e.g., specific age groups) for parameters associated with FH (e.g., abnormally high cholesterol levels) [6,13,14]. In both cases, these approaches would then lead to more or less systematic and centrally-coordinated cascade testing approaches in which at-risk family members of index cases would be identified and assessed [6].

Targeted screening approaches for FH have been implemented in several countries including the UK and the Netherlands. In these contexts, adults are identified by using systematic protocols such as the Simon-Broome and Dutch diagnostic criteria [10,11,12]. Strategies closer to universal screening have been applied in pre-school settings in Slovenia [13], and elementary- and middle schools in the United States (West Virginia, Utah, TX, USA) [14,15]. In West Virginia, for example, universal cholesterol screening of fifth graders in the state has been ongoing since 1998 (CARDIAC project) [14]. In Utah, high school students were engaged as part of classroom activities to collect their personal family history of CHD. Nurses were sent to do home assessments among families identified at highest risk [15].

Recently in the U.S., state departments of health have begun to consider universal FH screening efforts with children. In this report, we highlight the tensions and potential opportunities of universal screening. We draw on the experiences of the Netherlands, the UK and prior U.S.-based initiatives for issues that have arisen that could inform such programs. Specifically, we will: (1) briefly review the benefits of FH screening initiatives focusing on adults and what that tells us about screening children; (2) discuss the unique challenges and opportunities for universal FH screening of children; (3) describe the pros and cons of including genetic testing for children, and (4) make recommendations for how to implement programs that capitalize on the comparative strengths of clinical genetics, primary care and public health. The overarching aim of this report is to provide an overview of the considerations that should guide establishment of a screening program for FH.

### 1.1. Converging Social Forces Compel Universal Cholesterol Screening for Children

U.S. state health departments have begun to consider implementing universal FH screening programs via statewide cholesterol testing of children ages 9 to 11. Three key national recommendations are prompting this: (1) a 2011 National Heart, Lung, and Blood Institute (NHLBI) recommendation for universal lipid screening for those ages 9–12 that was then endorsed by the American Academy of Pediatrics [16]; (2) the Affordable Care Act that includes coverage of dyslipidemia screening as a preventive service for this age group, and (3) the Centers for Disease Control and Prevention’s (CDC) Office of Public Health Genomics (OPHG) evidence-based review that rated cascade testing for FH via cholesterol screening and DNA testing as a Tier 1 application, that is, ready for implementation. Additionally, other country’s success of implementing FH programs is motivating state programs to consider if and how screening might be implemented, focusing on children where opportunities for CHD prevention are greatest [17].

Among the many challenges to implementing such an ambitious endeavor will be identifying appropriate cholesterol levels for probable diagnosis and using family history screening to identify children at highest risk. Moreover, it is likely that technological advances and the decreasing costs of genetic analyses will compel consideration of expediencies that could be achieved by adding genetic testing in these programs. When health entities consider whether or not to use such innovations, the challenge will be to determine, for example, whether the value of genetic testing for more precisely characterizing high-risk children outweighs any potential downsides of such testing.

### 1.2. Aligning Stakeholders Representing Different Health Service Systems Will Be Key

A challenge for creating a population screening program for monogenic conditions, such as FH, is the need to engage stakeholders from different health service systems—clinical genetics, primary care and public health—that will be essential for coordinating follow-up and tracking high risk children and their families. These three systems have different operating practices and professional norms. For example, genetic clinics rely on referral and see individuals or families who have rare monogenic health conditions with multiple cases of the condition in the family. These practices tend to be based in high-risk clinics staffed by clinical geneticists and genetic counsellors. Typically, high-risk individuals are referred to the clinic and undergo genetic counseling and testing. Family pedigrees are generated and used to identify at-risk relatives who can subsequently be offered genetic counseling and testing if appropriate.

By contrast, primary care clinics provide acute care, chronic disease management and, increasingly, preventative care to a broad range of patients. In this setting, family history of common chronic disease and vital signs are routinely collected generally at clinic enrolment. However, this information is rarely used to identify common genetic disorders, such as FH [18]. This is due in part to health care providers’ lack of genetic knowledge and skills [19]. With an adequate family history taking around five minutes to complete, requirements to keep visit time short in primary care clinics is an additional barrier [20]. Most of the visit time is used to address the patients’ need for the current visit, often a health problem instead of a question about prevention. However, individuals with a raised cholesterol and family history of premature CHD seen in primary care can be identified. Similarly, clinicians managing care for an individual with myocardial infarction (MI) at an early age may also indicate FH [21]. Moreover, family history is increasingly being used for child health monitoring in the primary care setting, offering the opportunity to identify suspicion of FH in the extended family [22].

Public health organizations by contrast are charged with linking individuals and families to appropriate services to reduce risk factors as well as disease screening, primarily for common diseases, for large populations. These programs are generally based in health departments and agencies providing public health services, with professionals who often have little or no expertise in genetics. However, state public health programs historically have overseen population-based newborn screening [23] that is used to identify many genetic conditions by testing of metabolites, proteins or function (DNA tests are not often included). This information provided during the first days of a newborn’s life has critical importance for preventing severe health problems, including death and developmental delay. Moreover, beginning in 2003 in the U.S., the CDC began funding state health departments to develop statewide public health genomics programs with the purpose of integrating genomics into chronic disease programs where possible [24]. State genomics programs with an adult chronic disease focus have been successfully sustained primarily in the cancer realm due to national evidence-based recommendations for BRCA and Lynch syndrome and have begun to show evidence of benefits of cascade screening for adult relatives of individuals diagnosed with BRCA [25] and Lynch syndrome [26].

## 2. FH Screening Programs: Current Challenges and Opportunities

Whether it is advisable or not to initiate any screening program can be guided by the Wilson and Jungner criteria [27]. These criteria hold that the condition merits broad screening endeavors if it is an important health problem, the natural history of the condition is adequately understood, an accepted treatment is available, acceptable diagnostic tests are available, the cost of case-finding is economically in balance relative to medical expenditure.

FH screening meets most of these criteria. As a “common” monogenic condition associated with increased risk for CHD mortality, FH is clearly an important health problem. Lipid-lowering therapies, in particular statins, have dramatically improved life expectancy among adults [8]. Additionally, combined improvements in physical activity and diet, and avoiding tobacco use can lower risk of CHD among those with FH. The few intensive interventions evaluated to improve these multiple lifestyle factors among adults with FH have not shown significant improvements in reducing LDL levels over usual care. However, it is noteworthy that this finding was due in part to high baseline levels adherence to recommended levels of physical activity and not smoking among this high risk group [28]. There are acceptable and reliable screening tests available for adults to identify FH, and the cost-effectiveness of screening for FH in adults is recognized [29].

However, whether universal screening for children meets all of these criteria is a matter of continuing debate. There continues to be controversy regarding whether universal cholesterol screening could be used to identify FH risk among children. Additionally, the use, timing and safety of statin treatment, and the cost benefit of screening efforts are still not clear [30]. Overcoming these challenges, however, may be warranted by the strong case for primary prevention and potential for risk factor reduction early in life.

### 2.1. Challenges of FH Screening Approach Targeting Adults

Among adults, targeted screening approaches used to identify probable FH have had limitations. As stated earlier, rates of identification are generally low, as low as 15%. This may be due in part to the screening programs’ reliance on clinical settings to identify individuals that have limited reach. The direct family-tracing cascade genetic screening programs such as deployed in the Netherlands had greater success, particularly when accompanied by home visits for testing [31]. However, adult programs likely will continue to have incomplete reach due to lack of appropriate settings for identifying index patients.

In identifying adults with possible FH, an additional challenge is that cholesterol diagnostic cut offs become less precise with increasing age with the distribution of cholesterol levels for monogenic and polygenic hypercholesterolemia overlapping [32]. Additionally, cholesterol levels among relatives of confirmed FH cases vary widely by gender and age making it difficult to reach consensus on what constitutes an abnormally high cholesterol level. Accordingly, cut-off points used for such screening have varied widely. Thus, clinicians cannot rely on cholesterol alone for a definitive diagnosis of FH and must include family history and often clinical examinations [10,12].

DNA testing can augment efforts to confirm FH. However, there are a large number of mutations associated with FH and some mutations are more likely to result in FH than others [33]. Additionally, mutation patterns can differ between countries. This makes it difficult to select an optimal genetic testing platform. For example, the diversity of mutations makes conventional genetic testing too time-consuming and expensive. However, with the emergence of next-generation sequencing, the cost of testing a panel of genetic mutations is likely to decrease rapidly.

### 2.2. How Might Initiating FH Screening with Children Overcome These Challenges?

Universal screening of children for FH has been suggested as a means of overcoming some of the challenges experienced in using screening approaches with adults [14,34]. Potential advantages include: (1) the ability to target interventions early for prevention and treatment; (2) the availability of broad-based infrastructures that enable ready access to children and broader program reach; and (3) conceptual support that parents’ desire to protect children could extend intervention benefits to broader family networks. These compelling opportunities and the accompanying challenges for each are described in turn below.

#### 2.2.1. Ability to Target Interventions Early for Prevention and Treatment

Consideration of using a universal screening approach with children aligns with forecast advantages of precision medicine for prevention [35]. As the atherosclerotic process begins in childhood, opportunities to identify and offer treatments that could reduce related morbidity and mortality would be most beneficial when started early [17,30]. Indeed, in a large ongoing universal screening program of fifth graders in West Virginia, a third of parents reported making changes in children’s diets and physical activity levels when the child was found to have FH [36]. Additionally, these children were referred to a specialized children’s lipid clinic for regular intervention and follow-up as well as a statewide obesity intervention.

Evidence regarding the optimal age at which screening should be initiated with children is less clear. Prior screening experiences in the U.K. have been initiated at about age 10 [12], whilst in Slovenia universal screening is offered beginning at five years old [13]. In the Netherlands, children can undergo genetic testing beginning at age of six if one of the parent’s genetic test results is positive [37]. The lack of standardization in the age to initiate screening likely reflects the lack of consensus on the age to begin statin prescribing, usually around 8–10 years [17]. Currently, such practices are based on age of onset of CHD within the family and presence of other cardiovascular risk factors. Implementation of any new FH screening initiative involving very young children (e.g., under the age of the five) would benefit from efforts to understand the value added for prevention as well as acceptability to parents.

#### 2.2.2. Broad-Based Infrastructures for Accessing Children Could Extend Program Reach

Another benefit of using universal screening approaches to identify FH in children is the ready availability of access points that could overcome the limited reach of adult programs. For example, FH screening could be piggy-backed onto routine well-child visits in pediatric and family physician offices [13,16]. Potential opportune clinical portals include linking screening to school physicals and vaccinations. Wald and colleagues suggest a strategy in which children would be screened for FH at the time of vaccination occurring about 15 months of age by collecting a blood spot to use cholesterol testing to identify cases [38]. Universal screening in each of these settings raises numerous potential challenges relating to follow-up care and preventative strategies as well as consideration of ethical, legal and social implications.

School systems also would be obvious partners for universal screening. Indeed, in the last few decades, Utah and West Virginia both have conducted screening in high schools and elementary schools [14,15]. In each case children and families, a majority being white, have participated in relatively low cost take-home family history assessments. In both contexts, large numbers of school children have been engaged and these experiences largely have been successful. In Utah, 11% (2666) of family histories collected identified premature coronary heart disease or stroke in relatives [15], while in West Virginia, almost three quarters of children had a positive family history for CHD [14]. In West Virginia, the findings showed that reliance on family history as the screener would have missed children with moderate and genetic dyslipidemia whilst universal cholesterol screening would have identified all children with severe dyslipidemia [14]. From a recently published study, describing a national cholesterol screening program aimed at five-year old Slovenian children, it was also concluded that universal screening in children may be more reliable in identifying FH than selective screening based on family history [39].

#### 2.2.3. Screening Children Could Be Optimal for Extending Intervention Benefits to Broader Family Networks

Most prior screening programs have relied on targeted case finding among adults and then have identified other family members (including minor children) via cascade screening, a “top-down” approach. This screening approach has relied on the identified cases to pass on information directly to other family members. Even in the Netherlands with 20 years of direct cascade screening experience using genetic field workers to contact family members, over 50% of affected individuals were still not identified using this approach [31]. Universal screening of children, a “bottom-up” approach has some intuitive appeal. The thinking is that participation rates and adoption of risk-reducing behaviors among adults could be increased by leveraging the interests of their children’s health. There is strong conceptual rationale to support the notion that parents’ appraisals of their child’s health risks prompts risk-reducing actions for the child’s sake that parents have been unwilling to take on their own behalf. Moreover, reducing the child’s health risks also can be framed as a shared benefit, prompting “communal coping” amongst the broader family [40]. Thus, relatives may be more willing to become involved in further follow-up and preventive measures (such as diet and non-smoking advice). These bottom-up strategies also could offer the opportunity for lifestyle advice to be extended to other family members in the kinship network, even those at average risk, to create a supportive environment for the high-risk child. Indeed, it is widely agreed that most successful behavior change interventions for children are those that engage family [41]. Accordingly, Ritchie and colleagues describe efforts in West Virginia to involve families in weight loss efforts [14].

However, there are potential downsides to bottom-up universal screening that would then enable cascading up from the “healthy” child to adult relatives. For example, this approach could miss opportunities to intervene with adults before premature heart disease occurs. Further, bottom-up approaches could exacerbate previously raised concerns about undermining relative’s autonomy to decide about screening and their right ‘not to know’ their health status [42]. However, FH experiences from the Netherlands and the literature generally would suggest that most individuals were satisfied with the method by which they were approached [43].

## 3. Opportunities and Challenges of Including Genetic Testing in Screening Children for FH

As noted previously, reliance solely on phenotypic features such as cholesterol testing, family history assessments, and physical manifestations has resulted in a significant proportion of misidentification. This likelihood may be particularly consequential for individuals with polygenic disease [6,32] who are misidentified as having monogenic FH. Individuals with polygenic multifactorial FH have less severe prognosis and their relatives’ risk estimates are lower as well. For these cases, among adults, DNA testing has been regarded to be a more reliable and cost-effective method for diagnostically confirming cases of FH [44].

In the Netherlands, the cascade screening program was primarily designed for adults. However, the possibility of screening children was discussed with parents, and the number of children tested increased over the years. Findings from these efforts are consistent with an earlier study that showed the great majority of parents (87%) from FH families wanted their children to undergo a genetic test [45]. However, there is no evidence regarding how the broader population of parents (families at average risk for CHD) would feel about having their children tested as part of universal screening programs.

Reliance on phenotypes such as cholesterol cut-offs to diagnose FH in relatives introduces uncertainty for relatives. Genetic testing improves the accuracy of diagnosis and in so doing could exclude some from needed further monitoring. However, applications of genetic testing may intensify concerns raised above about universal screening such as undermining autonomy of relatives. Moreover, numerous unique concerns have been raised about downsides of genetic testing, particularly approaches such as whole genome sequencing. Notable among them is how to handle incidental findings, that is, findings that were not the focus of the genetic test but have health significance [46]. Thus, it will be important that consent processes provide sufficient information to families about these downsides.

With respect to using genetic testing with children, for most Western countries, genetic screening is an integral part of screening in early childhood through adoption of neonatal screening programs. Outside the neonatal period, genetic screening of minors is uncommon, in particular for adult-onset disorders. Most expert panels have recommended against testing in children until they are mature enough to understand the implications of testing [47]. With the pathological changes of FH emerging in early childhood, FH may be considered an exception. Interventions could begin earlier to promote a healthy lifestyle and statin treatment from as early as age 8–10 years [17,30]. This is consistent with the recent recommendation of the American Academy of Pediatrics that “Predictive genetic testing for adult onset conditions generally should be deferred unless an intervention initiated in childhood may reduce morbidity or mortality” [48].

Universal screening of children that includes genetic testing followed by cascade screening of other family members has several advantages. However, as with other monogenic conditions, such approaches do raise ethical concerns and challenges with respect to influences on communication (e.g., non-paternity) [49], interpersonal relationships (e.g., stigma towards children found to have disease) and psychological outcomes (e.g., labelled as high risk negatively influencing life goals) [13,50]. However, the limited research conducted to date has shown that children identified as FH mutation carriers generally cope well [51,52,53]. Thus, analysis of the potential impacts of such screening to reduce the severity of morbidity and improve long-term health may show that the benefits outweigh these risks.

## 4. Balancing Opportunities and Challenges of Screening Children for FH: Recommendations for a Way Forward

Using universal screening for FH as a means to reduce harm and ensure optimal treatment as early as possible could add decades of healthy life for those with FH [17]. Review of the experiences of targeted screening programs and early efforts at universal screening with children support these benefits but also present challenges that will need to be addressed. Moreover, challenges presented by misdiagnosis and steadily decreasing costs of genomic sequencing suggest that consideration should be given to incorporating genetic testing, using NGS techniques [39], in universal screening programs with children. These discussions must be informed by data regarding costs, measured health benefits, and ethical tradeoffs. Moreover, it should be noted that what works in one country may not work in others.

Universal screening of children also will require cooperation and coordination among clinical genetic specialists, primary care and public health providers to arrive at a care pathway for children found to have FH. Schools may be central in this endeavor. For example incorporation of family history recording of common inherited disorders into the school curriculum might be adopted to facilitate implementation of universal screening. Other modalities of community engagement, such as, consumer-friendly fact sheets, media campaigns and collaboration with the large community health work force [54]. As a chronic disease, FH universal screening also would require system coordination that maps a care pathway through the transition from pediatric to adult services. In parallel a strategy for identifying other relatives will be needed that too may involve these three systems.

## 5. Conclusions

Screening approaches for FH in the past showed incomplete identification of cases. Increasing genome technological capability will force a discussion of including genetic tests in these screening programs. The challenges for U.S. and other state departments of health will be to engage all key stakeholders including clinical genetic departments, primary care, lipid specialists as well as patient organizations [55]. Challenges also will be presented for facilitating the necessary shared vision for the optimal age of FH identification and the appropriate age at which to introduce statins in children diagnosed with FH. Using cholesterol cut-offs for screening will remain controversial, but resource limitations may justify its continued use. Additional information is needed to evaluate the cost-benefit of mounting universal screening for children’s health outcomes in the short- and long term.
